# A Multimodal Deep Learning Model for the Classification of Breast Cancer Subtypes

**DOI:** 10.3390/diagnostics15080995

**Published:** 2025-04-14

**Authors:** Chaima Ben Rabah, Aamenah Sattar, Ahmed Ibrahim, Ahmed Serag

**Affiliations:** 1 AI Innovation Lab, Weill Cornell Medicine, Doha 24144, Qatar; 2 Department of Medicine, New Vision University, 0159 Tbilisi, Georgia

**Keywords:** breast cancer, molecular subtype classification, deep learning, artificial intelligence, multimodal, personalized medicine

## Abstract

**Background**: Breast cancer is a heterogeneous disease with distinct molecular subtypes, each requiring tailored therapeutic strategies. Accurate classification of these subtypes is crucial for optimizing treatment and improving patient outcomes. While immunohistochemistry remains the gold standard for subtyping, it is invasive and may not fully capture tumor heterogeneity. Artificial Intelligence (AI), particularly Deep Learning (DL), offers a promising non-invasive alternative by analyzing medical imaging data. **Methods**: In this study, we propose a multimodal DL model that integrates mammography images with clinical metadata to classify breast lesions into five categories: benign, luminal A, luminal B, HER2-enriched, and triple-negative. Using the publicly available Chinese Mammography Database (CMMD), our model was trained and evaluated on a dataset of 4056 images from 1775 patients. **Results**: The proposed multimodal approach significantly outperformed a unimodal model based solely on mammography images, achieving an AUC of 88.87% for multiclass classification of these five categories, compared to 61.3% AUC for the unimodal model. **Conclusions**: These findings highlight the potential of multimodal AI-driven approaches for non-invasive breast cancer subtype classification, paving the way for improved diagnostic precision and personalized treatment strategies.

## 1. Introduction

Breast cancer is a heterogeneous disease with diverse molecular subtypes, each exhibiting distinct biological and clinical characteristics [1,2]. Accurate identification and classification of these subtypes are crucial for guiding tailored therapeutic interventions, improving patient outcomes, and optimizing treatment strategies. Breast cancer subtypes have been characterized based on gene expression patterns [3,4,5], including estrogen receptor (ER)-positive, progesterone receptor (PR)-positive, human epidermal growth factor receptor 2 (HER2)-positive, and triple-negative (TN) breast cancer, with each subtype requiring unique management approaches [4]. Early and accurate detection of these subtypes is essential for selecting the most effective treatment, minimizing unnecessary side effects, and ultimately improving patient outcomes. Among these, luminal A tumors are the most common and tend to have a better prognosis due to their responsiveness to hormone therapy. Luminal A tumors are ER-positive and typically respond well to endocrine therapies such as tamoxifen and aromatase inhibitors [6]. Luminal B tumors are also hormone receptor-positive but have higher proliferation rates and may require both endocrine therapy and chemotherapy [7].

In contrast, TN breast cancer, often associated with basal-like features, lacks ER, PR, and HER2 expression, making it unresponsive to hormone therapy or HER2-targeted treatments [8]. TN typically requires chemotherapy, but ongoing research explores the efficacy of immunotherapy [9]. HER2-enriched subtypes, while more aggressive, respond to targeted therapies such as trastuzumab (Herceptin). Hence, identifying subtypes early is vital for applying the correct treatment modality.

Molecular subtyping helps predict disease progression, recurrence risks, and overall survival [10]. For instance, luminal A tumors have a better prognosis with lower recurrence rates, while TN has a higher likelihood of early metastasis and poor survival outcomes. Identifying these subtypes early allows for better risk stratification and monitoring strategies. The identification of breast cancer subtypes can be achieved through several methods, including gene expression profiling, which classifies breast cancer into luminal A, luminal B, HER2-enriched, and basal-like subtypes. Although immunohistochemistry (IHC) remains the gold standard for breast cancer subtyping, it is not without limitations [11]. IHC requires invasive tissue biopsies, which may not fully capture tumor heterogeneity, especially in cases of metastasis or when the tumor exhibits spatial or temporal variations. Furthermore, biopsies may not always be feasible due to the location of the tumor, the patient’s condition, or other logistical constraints [12].

Recent advancements in medical imaging and Artificial Intelligence (AI) offer promising avenues for non-invasive breast cancer subtyping. Imaging modalities such as mammography, ultrasound, and magnetic resonance imaging (MRI) provide valuable insights into tumor characteristics. Radiomics, the extraction of quantitative features from medical images, has shown significant promise in correlating radiological characteristics with breast cancer subtypes [13]. MRI has been particularly valuable due to its ability to provide detailed tissue characterization, including information about tumor heterogeneity, vascularity, and tissue composition. Several studies have explored the association between radiologic features and breast cancer subtypes, with promising results indicating that certain imaging features may be linked to specific molecular subtypes [14].

However, the potential of digital mammography, a more widely accessible imaging modality, in this context remains less explored. Digital mammography offers essential insights into breast density, microcalcifications, and architectural distortions, all of which are important indicators of breast cancer. While it may not fully capture all tissue properties compared to MRI, it remains one of the most widely used screening tools due to its affordability and ease of access [15].

Deep learning (DL), a subset of AI, has shown great potential in extracting complex, high-dimensional features from medical images [16,17,18]. These algorithms can detect subtle patterns in images that facilitate classification into different categories, even when these patterns are imperceptible to the human eye [19]. DL has already proven successful in various applications of medical imaging, such as skin cancer detection and classification [20] and diabetic peripheral neuropathy detection [21], among others. By applying DL to digital mammograms, researchers can potentially identify correlations between image features and molecular subtypes, facilitating non-invasive subtype classification.

A growing body of research explores AI-driven approaches that integrate radiological data with clinical metadata to improve classification accuracy. Clinical factors such as patient age, hormone receptor status, and genetic information provide valuable context that enhances AI model performance. A multimodal approach combining radiological, clinical, and genomic data offers a more comprehensive assessment. Studies indicate that integrating imaging features with genetic and histopathological data strengthens predictive power for molecular subtypes. This fusion of multimodal data enhances prediction accuracy, particularly in diverse populations where imaging characteristics may vary due to genetic and environmental factors [22].

This study takes a novel approach by building on the work of Mota et al. [23], who demonstrated the potential of using AI based on mammography for molecular subtyping of breast cancer. We extend their approach by proposing a multimodal deep learning model that integrates image and metadata features, such as patient age and tumor class, for enhancing breast cancer subtype classification, while also embodying a hybrid approach through the combination of CNN-based feature extraction and feature fusion techniques. Age is a critical factor influencing breast cancer risk and progression, as highlighted by Mota and colleagues, who noted that younger women are more likely to develop aggressive subtypes like TN, while older women tend to develop hormone receptor-positive cancers. Tumor class, on the other hand, provides essential insights into the biological and molecular characteristics of the disease. By combining these clinical variables with image-derived features, our model aims to overcome the limitations of imaging-only approaches, such as the challenges posed by class imbalance and subtle differences between subtypes, as observed in the study by Mota et al. This integrated approach aims to improve prediction accuracy and provide a more comprehensive tool for personalized breast cancer diagnosis and treatment planning.

This study aims to leverage the power of AI to investigate whether clinically relevant breast cancer subtypes can be accurately classified using features extracted directly from digital mammograms, in conjunction with relevant clinical metadata. By analyzing a cohort of Chinese women using a publicly available dataset called the Chinese Mammography Database (CMMD), we aim to investigate how well various image features and clinical variables can be used to improve the classification of molecular subtypes.

## 2. Materials and Methods

### 2.1. Dataset

This study uses CMMD [24], a collection of breast mammography images and the corresponding clinical data for the subjects. Both the images and data are publicly available in The Cancer Imaging Archive (TCIA) at www.cancerimagingarchive.net (accessed on 5 February 2025). The image data underwent standard processing procedures as outlined by TCIA’s curation workflows. Importantly, TCIA employs a rigorous, standards-based approach to anonymize all images stored in the Digital Imaging and Communications in Medicine (DICOM) format.

**Figure 1:**
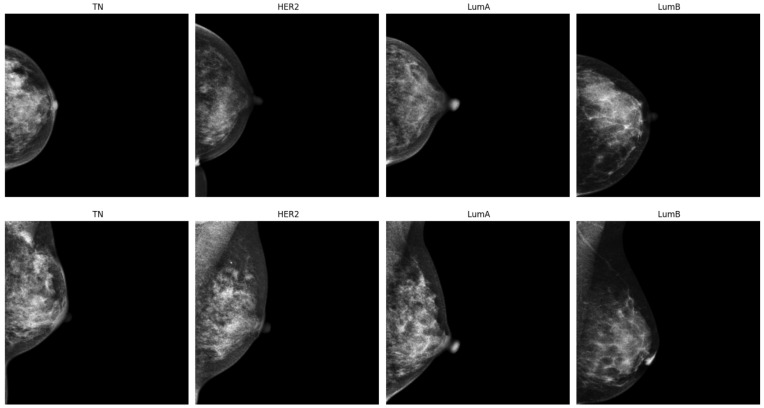
Illustration of two samples of each molecular subtype: TN, HER2, luminal A, and luminal B.

The CMMD dataset consists of 5202 mammography images of size 2294 × 1914 and 1873 clinical data collected between July 2012 and January 2016 from 1775 patients in China. The images are categorized into five classes: benign, HER2, luminal A, luminal B, and TN. Samples representing each molecular subtype are shown in Figure 1.

**Figure 2:**
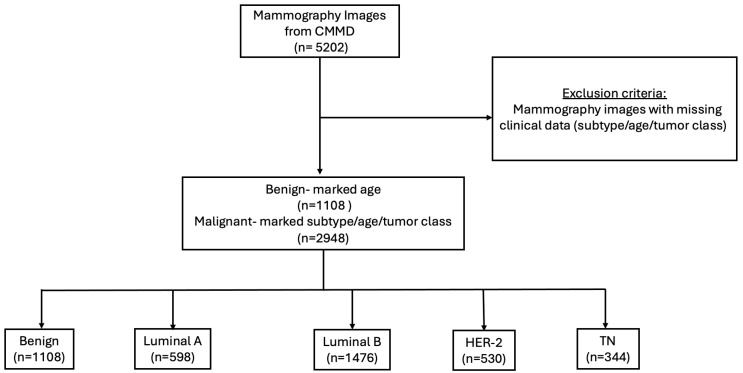
Flowchart of mammography images inclusion and exclusion criteria.

We considered only images with complete clinical data (age and calcification type) and information about the molecular subtype (if not benign). For further details regarding the patient inclusion and exclusion criteria employed in this study, please refer to Figure 2.

### 2.2. Data Preprocessing

Image preprocessing is a critical step in the analysis of medical images. By enhancing image quality and accuracy, preprocessing significantly impacts the reliability of subsequent analyses, ultimately contributing to improved diagnostic and therapeutic outcomes. In our approach, the mammography images were resized to 224 × 224 pixels to match the input size required by pre-trained CNN models which were originally trained on ImageNet [25]. This resizing ensures compatibility and takes advantage of transfer learning. After resizing, the images were normalized to a range of [0, 1]. This normalization step helps improve the stability of the model and accelerates the convergence during training.

**Figure 3:**
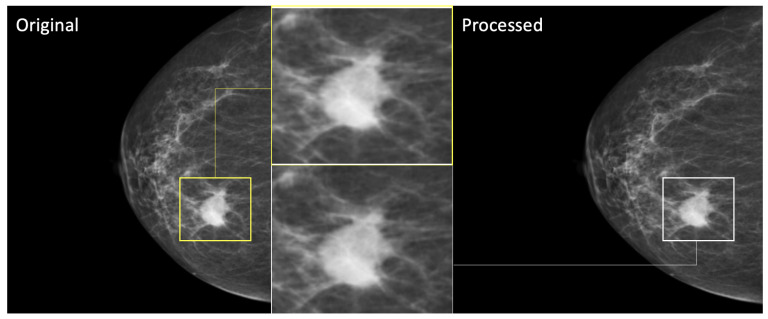
Comparison of a lesion before and after resampling. On the **left**, the original high-resolution image of the lesion. On the **right**, the lesion after resampling to the target resolution (224 × 224). In the center, a zoomed-in view of the lesion is provided for each image, highlighting the preservation of key features and structural details following resampling.

To verify that resampling did not compromise the quality of the lesions, we conducted a visual comparison of the original and resampled images. As shown in Figure 3, the zoomed-in view of a representative lesion confirms that resampling preserved the structural integrity and key features, with no significant loss of diagnostic information.

**Figure 4:**
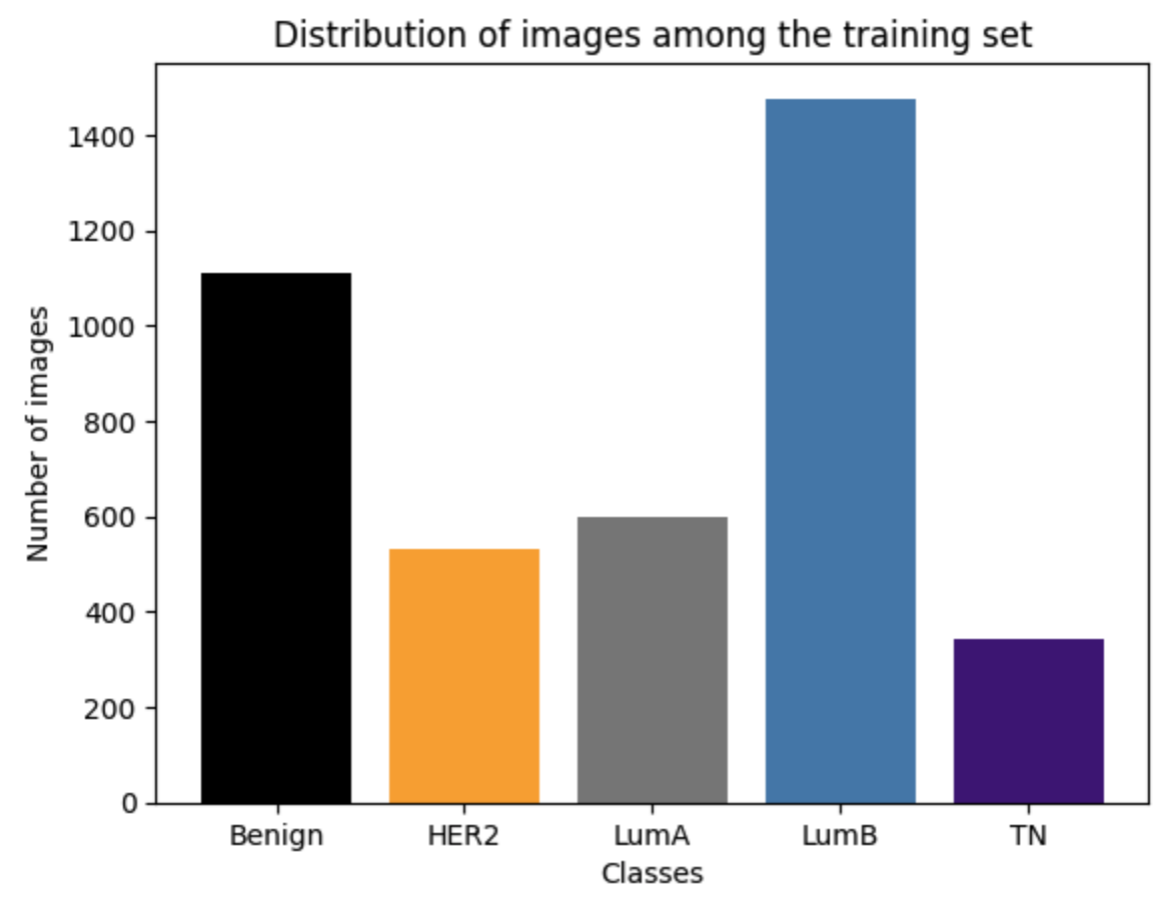
Distribution of the molecular subtypes among the training set.

Class imbalance, where certain classes are underrepresented, can lead to biased model performance, as the algorithm may favor the majority class and underperform on minority classes. Figure 4 illustrates the class distribution in the training set before balancing. To mitigate this, we calculated the class weights inversely proportional to the frequency of each class in the training data, ensuring that the model paid more attention to underrepresented classes. These weights were incorporated into the loss function, penalizing misclassifications of minority classes more heavily. This approach helped improve the model’s ability to generalize across all classes, particularly in medical applications where accurate classification of rare but clinically significant cases is critical.

### 2.3. Multimodal Model

**Figure 5:**
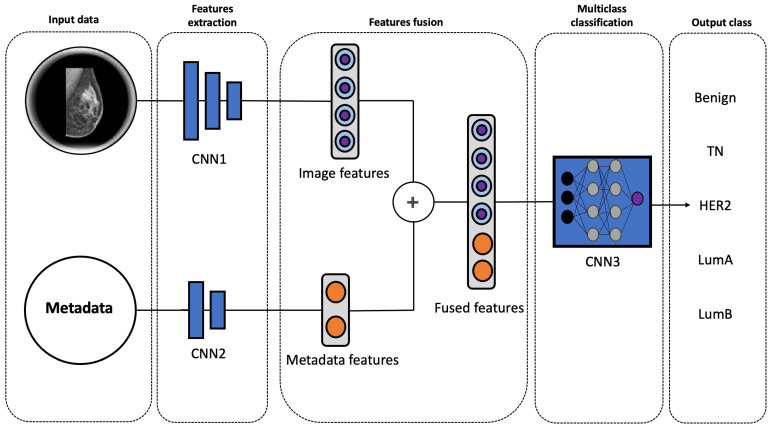
Architecture of the proposed multimodal model. CNN1, CNN2, and CNN3 represent distinct convolutional neural networks. The “+” symbol denotes the concatenation operation.

Figure 5 illustrates the architecture of the proposed multimodal model. The network comprises three distinct components, all based on Convolutional Neural Networks (CNNs): two encoders (CNN1 and CNN2) and a classification network (CNN3).

The first encoder extracts high-level features from the input images, while the second encoder processes the associated metadata. The extracted features are then fused into a unified representation, capturing the combined information from both modalities.

The fused representation is subsequently fed into the classification network, which generates the final predictions.

To extract high-level image features, CNN1 utilizes a pre-trained Xception model, an advanced architecture inspired by Inception-V3 [26]. Given the limited size of our dataset and the data-intensive nature of the Xception model, leveraging a pre-trained model on ImageNet is crucial for achieving optimal performance. To enhance feature extraction, the pre-trained Xception model is fine-tuned with additional layers, including a global average pooling layer, a dense layer, a ReLU activation function, and a dropout layer. This process yields a feature vector of size 512.

In parallel, the metadata associated with each image are processed by a dedicated encoder, CNN2. Age is binarized into two categories: 0 for patients under 40 years of age and 1 for those 40 years and older. This threshold is widely used in clinical and research settings, as age 40 is a significant milestone in breast cancer screening guidelines [27,28] and reflects increased risk and biological differences in tumor characteristics [29,30]. The tumor class is categorically encoded as follows: 0 for none, 1 for mass, 2 for calcification, and 3 for both. The encoded metadata is then fed into a neural network consisting of a dense layer with ReLU activation followed by a dropout layer. The output of this network is a feature vector of size 32, designed to match the dimensionality of the image feature vector extracted by CNN1.

Finally, the extracted image features (size 512) and encoded metadata features (size 32) are fused using a simple concatenation operation, resulting in a unified representation of size 544. This fused representation, which encapsulates information from both modalities, is subsequently fed into the CNN3 classification network. CNN3 comprises three dense layers with ReLU activation functions, culminating in the final classification predictions.

### 2.4. Implementation Details

The cleaned dataset consists of 4056 mammography images with associated metadata. To facilitate model training, validation, and testing, the dataset was divided into three subsets using an image-wise split, a common practice in similar studies [23]. This approach was adopted to address the limited number of patients in certain subtypes, particularly the TN class.

The dataset was partitioned as follows: the training set (72%, 2920 images), the validation set (18%, 730 images), and the test set (10%, 406 images). This split ensures a balanced distribution of data across all phases, enabling robust model training, hyperparameter tuning, and unbiased evaluation. To ensure a representative distribution of classes in each set, the dataset was split using stratified sampling based on class labels. This approach guarantees that the proportion of each class in each set mirrors the proportion in the original dataset, mitigating potential class imbalance issues and ensuring fair model evaluation.

To demonstrate the impact of incorporating clinical data, we also implemented a unimodal approach that relied solely on imaging data. In this approach, imaging features were extracted using CNN1 and subsequently classified using CNN3. The unimodal system was trained, validated, and tested on the same dataset used for the multimodal approach to ensure a fair comparison.

All experiments were conducted on a single GPU (NVIDIA A100 80GB) with a batch size of 64. The models were trained for 10 epochs to prevent overfitting. The weighted cross-entropy loss assigns a weight to each category to handle class imbalance. For optimization, we used the Adam optimizer with a learning rate of 0.001.

To promote transparency and reproducibility, we have made our code available on GitHub. The repository can be accessed at https://github.com/serag-ai/Multimodal-BC-Classifier (accessed on 5 February 2025).

### 2.5. Evaluation Metrics

The model’s classification performance was assessed using standard metrics: accuracy (ACC), precision (PRE), recall (REC), receiver operating characteristic (ROC) curve, and area under the curve (AUC). Given the imbalanced nature of the dataset, the F1 score was used as a robust metric to mitigate the potential biases of other metrics. All metrics were reported as percentages.

## 3. Results

**Figure 6:**
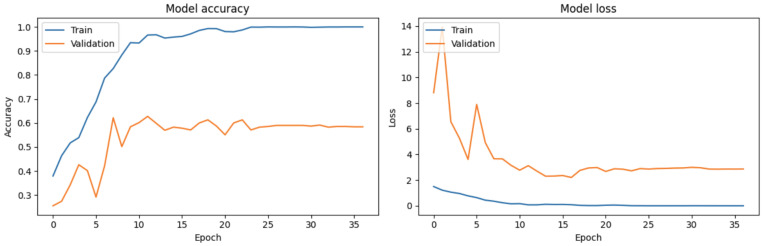
The evolution of accuracy (**left**) and the loss function (**right**) over the epochs for the multimodal model.

The multimodal model converged quickly and achieved an optimal AUC in a few epochs. Figure 6 shows the AUC and loss values over the number of epochs. It is evident that after epoch 10, the model began to overfit, as indicated by the divergence between training and validation performance. To avoid overfitting and ensure optimal generalization, we decided to stop further training at this point.

**Figure 7:**
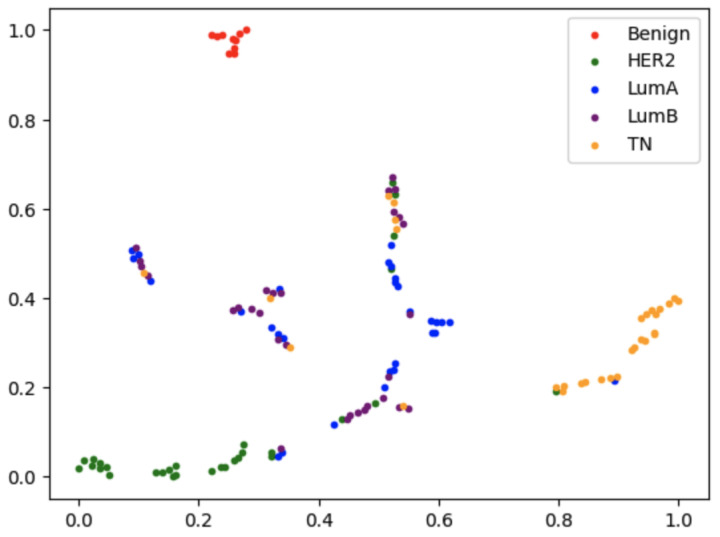
t-SNE visualization of the data distribution for breast cancer subtypes. The plot shows the 2D embedding of the high-dimensional feature space, highlighting the separation between different classes: benign, HER2, luminal A, luminal B, and TN. Each point represents a sample, and its color corresponds to the class label. For visualization purposes, only 20 randomly selected samples per class are shown.

To gain deeper insights into the distribution of breast cancer subtypes in the feature space, we applied t-SNE (t-Distributed Stochastic Neighbor Embedding) to visualize the high-dimensional data in a 2D space. As shown in Figure 7, the t-SNE plot reveals the clustering patterns of different classes, including benign, HER2, luminal A, luminal B, and TN. Each class is represented by a different color. The visualization demonstrates varying degrees of separation between subtypes. For example, HER2 and TN samples tend to form different clusters, indicating that these subtypes have unique feature representations that are easier to differentiate. In contrast, luminal A and luminal B samples show more overlap, reflecting the challenges in distinguishing these subtypes due to their similar imaging characteristics. The t-SNE plot provides valuable insights into the intrinsic structure of the data and highlights the challenges in subtype discrimination, particularly for luminal A and luminal B. These findings underscore the need for further refinement of the feature extraction and classification strategies of these subtypes.

The Xception model integrated into our framework achieves significantly superior performance compared to other widely used CNN backbones. To validate this, we performed an extensive comparison with state-of-the-art models such as InceptionV3, EfficientNetB7, ResNet50, and VGG16. As shown in Table 1, the proposed framework, leveraging Xception, consistently outperforms these models across all evaluation metrics.

A comparative experiment between the unimodal and the multimodal approaches in classifying molecular subtypes and benign cases is presented in Table 2. The results indicate a clear advantage for the proposed multimodal model. It exhibited superior performance across all evaluation metrics, achieving an accuracy of 63.79% and an AUC of 88.87%, compared to the unimodal model trained on mammography images alone, which yielded an accuracy of 31.78% and an AUC of 61.3%. It is clear that the unimodal model exhibited limitations in capturing the full complexity of breast cancer molecular subtyping, particularly in cases where imaging features alone were insufficient to distinguish between subtypes with overlapping morphological characteristics. This underscores the importance of combining imaging data with clinical metadata to enhance the predictive power of the model and to achieve more robust and accurate subtype classification.

**Figure 8:**
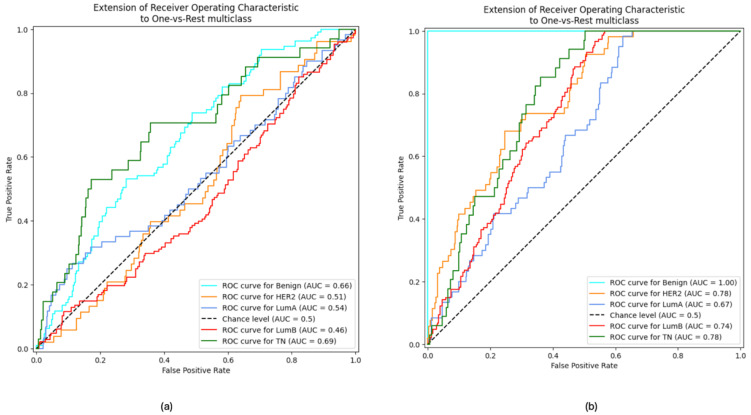
Comparison of ROC curves in the (**a**) unimodal approach and (**b**) multimodal approach.

While Table 2 summarizes the performance of the multiclass classification for both unimodal and multimodal models, Figure 8 illustrates the results of binary one-vs.-all classification, where each subtype is compared against all others. This binary approach provides additional insights into the model’s ability to distinguish individual subtypes from the rest.

Figure 8a,b shows one-vs.-rest ROC curves, where each curve represents the model’s performance in classifying a single class against all other classes. The AUC value for each curve reflects the model’s discriminative ability for that specific class. In Figure 8a, the ROC curve for TN has the highest AUC (69%), indicating the model is most effective at distinguishing TN breast cancer from other subtypes. However, luminal A and luminal B have the lowest AUC (54% and 46%), suggesting that the model struggles to accurately classify these subtypes compared to others. This observation aligns with the classification results presented earlier in Figure 7, where luminal subtypes overlap. It is also clear that the low AUC values for HER2, Luminal A, and Luminal B suggest that the model may have inherent biases or limitations in classifying these subtypes accurately.

Regarding the multimodal approach, it can be seen from Figure 8b that the AUC value of each category reaches about 60% or higher. Moreover, the ROC curves show that the classification of benign cases has the highest AUC (100%), indicating the model is most effective at distinguishing benign breast cancer from other subtypes. Conversely, luminal A and luminal B have the lowest AUCs (67% and 74%), suggesting that the model still struggles to accurately classify luminal A and luminal B subtypes though with marked improvement compared to the unimodal approach.

To assess the impact of image resizing on model performance, we trained and tested our model using larger image sizes, specifically 512 × 512 and 1024 × 1024. The objective of this evaluation was to determine whether reducing the resolution of the images led to the loss of critical features necessary for an accurate classification. Our findings indicate that the model achieved an accuracy of 54.68% on 512 × 512 images and 43.6% on 1024 × 1024 images, compared to its baseline performance on 224 × 224 images. The decline in accuracy with increasing resolution suggests that the model may not be utilizing the additional information present in higher-resolution images effectively. This is largely attributable to the fact that increased complexity leads to overfitting. These findings emphasize the importance of selecting an appropriate input size to balance computational efficiency and feature retention in DL-based image classification tasks.

Compared to related work, Mota et al. [23] achieved approximately 62% accuracy in classifying molecular subtypes using only mammography images, as shown in Table 3. Their study relies solely on imaging data, and their results were lower than those of our multimodal model. In binary classification, our model achieves an AUC of 78% for TN vs. non-TN, surpassing the 64.45% AUC reported by Mota and colleagues, with data augmentation and oversampling. Similarly, for HER2 vs. non-HER2, we achieve an AUC of 78%, compared to their 73.31% AUC with data augmentation and undersampling. However, these comparisons should be interpreted with caution, as the class definitions and dataset composition differ between the two studies.

While our work includes benign tumors and four molecular subtypes, Mota et al. [23] focuses on luminal A, luminal B1, luminal B2, HER2, and TN. Additionally, they benefit from precise tumor localization, as their dataset provides the coordinates of the region of interest (ROI) around the tumor, allowing their model to focus exclusively on the tumor area. In contrast, our study does not have access to such precise localization information, which may introduce additional complexity into the classification process. Despite these differences, our multimodal approach, which incorporates clinical metadata, demonstrates superior performance compared to Mota et al., highlighting the added value of integrating clinical data with imaging features [22,31].

## 4. Discussion

This study introduces a novel multimodal approach for breast cancer subtype classification, integrating clinical metadata with mammography images within a DL framework. To the best of our knowledge, this is the first application of such a strategy in this context. Using a dataset of 4056 images from 1775 patients, our model demonstrated significant improvements over traditional unimodal approaches, achieving an accuracy of 63.79% and an AUC of 88.87%. Notably, the incorporation of clinical metadata, specifically age and lesion type, proved instrumental in enhancing the model’s ability to differentiate between subtypes, as well as between benign and malignant cases.

Prior research has underscored the relevance of clinical factors such as age and lesion characteristics in breast cancer diagnosis and prognosis [32]. Our results reinforce these findings by showing that these variables provide essential context that imaging alone cannot capture. Although the multimodal approach improved classification performance across most subtypes, distinguishing between luminal A and luminal B remained challenging. This difficulty, widely recognized in the literature [33,34], is attributed to the close biological resemblance between these ER-positive subtypes. The current study suggests that further enhancements may require the integration of additional data types, such as genetic markers and more detailed clinical information.

Our comparative analysis between unimodal (images only) and multimodal models revealed the substantial benefits of integrating clinical metadata. The unimodal model achieved an accuracy of 31.78% and an AUC of 61.3%, far below the performance of the multimodal model. The ROC curves (Figure 8) further illustrate these findings: while the unimodal approach was relatively effective in classifying TN breast cancer (highest AUC of 69%), it performed poorly in differentiating luminal subtypes (AUC values of 54% for luminal A and 46% for luminal B), which is notable in Figure 7. In contrast, the multimodal model improved AUC values across subtypes, most notably achieving an AUC of 100% for benign classifications, and thus demonstrated a clear advantage in sensitivity and specificity.

The enhanced performance of our multimodal model underscores the importance of incorporating clinical context into AI-driven diagnostic tools. The combination of imaging data and patient information not only improves overall classification metrics (accuracy, AUC, F1 score, precision, and recall) but also aligns with previous studies that reported improved diagnostic outcomes when clinical variables were included [35,36,37]. Specifically, our results demonstrate that incorporating metadata (age and tumor class) significantly improves the model’s performance, achieving an F1 score of 52%, precision of 46%, and recall of 64%, compared to 26%, 26%, and 29%, respectively, for images alone. This highlights the value of leveraging clinical data to enhance subtype classification. However, it is important to acknowledge that these performance metrics, while improved, remain low in absolute terms. This reflects inherent challenges in breast cancer subtype classification, such as subtle and overlapping mammographic features across subtypes, class imbalance, and the model’s potential reliance on global patterns rather than lesion-specific details. These limitations underscore the complexity of the task and the need for further research to improve model performance, potentially through more sophisticated architectures, additional data sources, or advanced techniques to address class imbalance and feature extraction.

The observed decline in model performance with increasing image resolution (from 224 × 224 to 512 × 512 and 1024 × 1024) highlights an important consideration in the classification of DL-based breast cancer subtypes. While higher-resolution images theoretically provide more detailed information, such as microcalcifications and fine-grained textures, our results suggest that the model struggles to leverage these additional details effectively. This counterintuitive outcome can be attributed to several factors. First, higher-resolution images introduce greater computational complexity and a larger parameter space, which can exacerbate overfitting, particularly when the training dataset is limited. Second, the model’s architecture may not be optimized to capture and interpret fine-grained features at higher resolutions, leading to suboptimal feature extraction. Moreover, the increased memory and processing demands may constrain the model’s ability to generalize effectively. These findings underscore the importance of balancing resolution with computational efficiency and model capacity. Future work could explore advanced architectures, such as attention mechanisms or multi-scale feature extraction, to better utilize high-resolution data while mitigating overfitting and computational bottlenecks. This would enable more effective exploitation of critical fine details for accurate classification of breast cancer subtypes.

The wide age range of our dataset (17–87 years) introduces variability in breast density, which is a known factor influencing mammographic imaging and breast cancer classification. Younger women typically have denser breast tissue, characterized by a higher proportion of glandular and fibrous tissue, which can obscure tumors and make classification more challenging [38,39]. This is reflected in the performance of our unimodal model, which achieved an AUC of only 61.3% when relying solely on mammography images. However, the inclusion of age and lesion type in the multimodal approach significantly improved performance, with an AUC of 88.87%. This suggests that age-related factors, including breast density, play a critical role in accurate classification. While the OPTIMAM dataset starts at 50 years, our dataset includes patients as young as 17 years, introducing additional complexity due to denser breast tissue. Despite this, our multimodal approach, which incorporates clinical metadata and benign cases, demonstrates broader applicability and better alignment with real-world clinical scenarios. Future work will explicitly incorporate breast density information to further enhance model performance and address the challenges posed by dense breast tissue.

Our approach demonstrates significant improvements over previous methods, such as the one proposed by Mota et al. [23], achieving an AUC improvement of over 28% and expanding the model to include benign tumors. However, direct comparisons with Mota and colleagues’ study are challenging due to the distinct characteristics of the datasets used in the two studies. The OPTIMAM dataset, utilized by Mota et al., is a private dataset with restricted access, containing over 3 million images from more than 172,000 patients, making it significantly larger and more diverse than the CMMD dataset. Moreover, they selected a subset of 1397 images from 660 patients for their study, making it difficult to ensure a fair evaluation using the same subset. Furthermore, Mota and colleagues leveraged precise tumor localization (region of interest coordinates) provided by the OPTIMAM dataset, a feature unavailable in the CMMD dataset. Additionally, discrepancies in class definitions and metadata (e.g., molecular subtypes, clinical variables) between the two datasets further complicate direct comparisons. These factors highlight the need for careful consideration when benchmarking across datasets with differing characteristics.

Despite promising results, several limitations should be addressed in future research. First, the dataset used in this study was limited to a cohort from a single center in China, which may restrict the generalizability of our findings. Future studies should include more diverse populations to evaluate the model’s robustness across different geographic and ethnic groups.

Second, the clinical metadata were restricted to age and tumor class. Although age is a well-established risk factor for breast cancer [40], its direct relationship with specific molecular subtypes remains unclear. Some studies suggest age-related differences in the prevalence of subtypes, with younger women more likely to develop breast cancer positive for TN or HER2 [41,42]. However, the biological mechanisms underlying these differences are not fully understood, making age a potentially less specific predictor in subtype classification models. Similarly, the type of calcification is an important imaging feature associated with malignancy [43], but its role in distinguishing molecular subtypes remains uncertain. Although some studies have investigated potential associations between calcifications and aggressive tumor subtypes [44,45,46], these findings have not been widely validated. Future research should critically evaluate the contribution of these metadata features. Incorporating additional clinical and genetic factors, such as circulating tumor DNA (ctDNA), family history, molecular biomarkers, and transcriptomic data, could refine the predictive power of AI-driven models [47]. Multi-omics integration, which combines genomics, transcriptomics, proteomics, and radiomics, has been shown to improve cancer classification and prognosis prediction [48,49,50]. By incorporating non-imaging data, we can gain deeper insight into tumor heterogeneity and response to treatment.

Additionally, the ongoing challenge of distinguishing luminal A from luminal B indicates that even more granular data may be required. Another limitation is the sole reliance on digital mammography. Other imaging modalities such as MRI and ultrasound could provide complementary information, particularly in dense breast tissue [51,52,53,54]. Studies have shown that multimodal imaging can improve breast cancer detection and classification by providing complementary information about tumor morphology and molecular characteristics [55]. Comparative studies utilizing various imaging techniques, as well as exploring advanced DL architectures (e.g., Xception networks, attention mechanisms, multitask learning, etc.), may further optimize model performance.

Furthermore, federated learning has emerged as a transformative approach for training multi-institutional AI models, enabling collaboration between healthcare institutions while preserving patient privacy and data security [56]. By allowing models to be trained on decentralized datasets without sharing raw data, federated learning addresses critical challenges related to data privacy regulations and institutional barriers. This approach not only facilitates the development of more diverse and representative models but also enhances generalizability across different populations and imaging protocols. Future research could explore advanced federated learning frameworks, such as adaptive aggregation techniques and differential privacy, to improve model performance and scalability in real-world settings.

In addition to technical advancements, clinical validation through large-scale, multicenter prospective trials will be essential to translate AI-driven breast cancer subtyping into routine clinical practice. Although retrospective studies have demonstrated the potential of AI in oncology, real-world validation is necessary to ensure the reliability, robustness, and clinical applicability of the model [57]. Prospective trials should focus on the evaluation of AI systems in various clinical settings, assessing their impact on diagnostic precision, treatment decision-making, and patient outcomes. In addition, integrating AI tools into clinical workflows will require addressing practical challenges, such as interoperability with existing healthcare systems, user interface design, and clinician trust in AI recommendations.

Collaborative efforts between radiologists, oncologists, pathologists, and data scientists will be critical in developing user-friendly, interpretable AI systems seamlessly integrated into routine diagnostics [58]. Interdisciplinary collaboration can ensure that AI models are clinically relevant, align with oncological guidelines, and provide actionable insights for personalized treatment planning. Furthermore, explainable artificial intelligence (XAI) techniques, such as attention mechanisms and saliency maps, can enhance the interpretability of the model, allowing clinicians to understand and trust AI-driven predictions. By fostering transparency and accountability, XAI can bridge the gap between AI research and clinical adoption.

By addressing these research directions, ranging from federated learning and prospective validation to interdisciplinary collaboration and explainability, future studies can refine AI-assisted breast cancer subtyping and overcome existing barriers to implementation. These advances have the potential to revolutionize oncology by enabling earlier and more accurate diagnoses, optimizing treatment strategies, and ultimately improving patient outcomes. As AI continues to evolve, its integration into precision medicine will pave the way for more personalized and effective cancer care, transforming the landscape of breast cancer management.

## 5. Conclusions

In summary, our multimodal AI model enhances breast cancer subtype classification by integrating mammography images with clinical metadata. This approach improves diagnostic accuracy and clinical relevance while establishing a foundation for future research on personalized breast cancer diagnosis. Further advances, including the incorporation of additional clinical and genetic factors, comparison of multiple imaging modalities, and the exploration of advanced deep learning architectures, will be crucial for refining these tools. Ultimately, these efforts aim to improve patient outcomes and enable more personalized treatment strategies.

## Figures and Tables

**Table 1:** Comparison of different CNN backbones used in our multimodal model.

	ACC	AUC	F1 Score	PRE	REC
VGG16	45.07%	84.45%	33%	29%	49%
ResNet50	46.31%	83.63%	38%	33%	49%
EfficientNetB7	45.07%	84.57%	33%	29%	49%
InceptionV3	47.78%	85.87%	38%	42%	46%
**Xception (proposed)**	**63.79%**	**88.87%**	**52%**	**46%**	**64%**

**Table 2:** Performance metrics for predicting breast cancer molecular subtypes using mammography images alone versus a multimodal approach that includes age and lesion type metadata.

	ACC	AUC	F1 Score	PRE	REC
Mammography Images Only	31.78%	61.3%	26%	26%	29%
**Mammography Images + Metadata**	**63.79%**	**88.87%**	**52%**	**46%**	**64%**

**Table 3:** A performance comparison between our AI system and a state-of-the-art study.

	Proposed Multimodal Approach	Mota et al. [23]
Dataset	CMMD	OPTIMAM
Number of images	4101	1397
Number of patients	1750	660
Patients’ age	17–87	50–90
Clinical metadata	✓	X
Benign class	✓	X
AUC	88.87%	60.62%

## Data Availability

The data used in this study are publicly available in The Cancer Imaging Archive (TCIA) at https://www.cancerimagingarchive.net/ (accessed on 5 February 2025). The source code will be shared upon acceptance of this paper.

## Abbreviations

The following abbreviations are used in this manuscript:

AI	Artificial Intelligence
ACC	ACCuracy
AUC	Area Under the ROC Curve
CNN	Convolutional neural networks
CMMd	Chinese Mammography Database
DICOM	Digital Imaging and Communications in Medicine
DL	Deep Learning
ER	Estrogen Receptor
HER2	Human Epidermal growth factor Receptor 2
IHC	ImmunoHistoChemistry
MRI	Magnetic Resonance Imaging
PR	Progesterone Receptor
PRE	PREcision
REC	RECall
ROC	Receiver Operating Characteristic
TCIA	The Cancer Imaging Archive
TN	Triple Negative
